# Load-sensitive impairment of working memory for biological motion in schizophrenia

**DOI:** 10.1371/journal.pone.0186498

**Published:** 2017-10-13

**Authors:** Hannah Lee, Jejoong Kim

**Affiliations:** Department of Psychology, Duksung Women’s University, Seoul, Republic of Korea; Vanderbilt University, UNITED STATES

## Abstract

Impaired working memory (WM) is a core cognitive deficit in schizophrenia. Nevertheless, past studies have reported that patients may also benefit from increasing salience of memory stimuli. Such efficient encoding largely depends upon precise perception. Thus an investigation on the relationship between perceptual processing and WM would be worthwhile. Here, we used biological motion (BM), a socially relevant stimulus that schizophrenics have difficulty discriminating from similar meaningless motions, in a delayed-response task. Non-BM stimuli and static polygons were also used for comparison. In each trial, one of the three types of stimuli was presented followed by two probes, with a short delay in between. Participants were asked to indicate whether one of them was identical to the memory item or both were novel. The number of memory items was one or two. Healthy controls were more accurate in recognizing BM than non-BM regardless of memory loads. Patients with schizophrenia exhibited similar accuracy patterns to those of controls in the Load 1 condition only. These results suggest that information contained in BM could facilitate WM encoding in general, but the effect is vulnerable to the increase of cognitive load in schizophrenia, implying inefficient encoding driven by imprecise perception.

## Introduction

Schizophrenia is a complex and severe mental disorder that affects about 1% of the population worldwide. In addition to the most prominent clinical features, including hallucinations, delusions, thought disorders, and flat affect [1], a wide range of cognitive deficits, such as attentional problems [2], impaired working memory (WM) [3–7], and abnormal executive functioning [8], also characterize the disorder. These cognitive deficits are important, as they affect sufferers’ social problem-solving abilities and disturb everyday life [9–12].

Among the various cognitive deficits, WM deficit is considered a cardinal feature of schizophrenia [13]; the majority of patients with schizophrenia show stable WM deficits [14] across diverse modalities and methods [5]. Because WM is a complex system that comprises various sub-processes [15], large numbers of studies have been conducted from diverse points of view to obtain a better understanding of the nature of WM impairments in schizophrenia. Considerable evidence points to impairments in WM maintenance [6, 16]. On the other hand, WM impairments also occur in tasks with relatively short delays [17, 18], which implies impairments at the earlier encoding stage. In general, the selection of task-relevant information during perceptual processes is critical for successful encoding, and this selection process can be bottom-up based on perceptual features of the target, top-down driven by knowledge and expectations, or both. Although patients may exhibit difficulty in the spontaneous top-down selection of task-related information for encoding, they seem to benefit to some extent from the salience of the stimuli aiding the bottom-up process. Indeed, there is evidence that increasing the perceptual salience of the stimuli facilitates the encoding process, resulting in improved visual WM performance in not only non-clinical individuals but also patients with schizophrenia [19–23]. For example, patients with schizophrenia exhibited improved performance on the Continuous Performance Task when the cue stimuli were presented with different colors (i.e., increased salience) [20]. When familiar stimuli (“A”s) were used, participants remembered the novel stimuli (inverted A) better than the familiar stimuli (upright A) [21]. Faces, very salient and socially meaningful stimuli, also lead to better WM performance than plain stimuli for both healthy individuals and patients with schizophrenia [22]. These past studies showed that patients with schizophrenia as well as healthy individuals benefit from apparently salient stimuli. However, to our knowledge, little research has been conducted examining how the WM-facilitating effect led by bottom-up cues is modulated for the stimuli for which patients experience difficulty in perceptual processing. As the encoding process seems to be largely dependent upon the efficient and appropriate processing of the stimuli, an investigation of the relationship between perceptual processing and WM in schizophrenia is worthwhile to elucidate their abnormality.

Another well-known deficit in schizophrenia related with the perceptual processing is visual dysfunction mainly associated with the function of the dorsal visual pathway [24–27]. For example, patients with schizophrenia exhibit a significantly longer-lasting visual backward masking effect compared to healthy people [24, 28–31]. They are also poor at discriminating motion velocity [25] and detecting global, coherent motion from noise elements [26, 32, 33]. These perceptual deficits could eventually interrupt pertinent behavioral responses in social situations, as suggested by the significant correlation with measured social functioning [34–36].

A decade ago, it was reported that patients with schizophrenia also exhibit impaired processing of a unique, socially relevant motion signal: biological motion (BM) [37, 38]. In general, people are highly adept at recognizing BM. Even when BM is portrayed by only a dozen point-lights (PLs) [39] on the major joints of the body, observers not only recognize it almost effortlessly [40, 41] but also are able to perceive socially meaningful information, such as gender and mood [42–44]. In contrast, patients with schizophrenia do not perform as skillfully as healthy people do on visual tasks using BM. According to past studies, even though the ability to recognize BM per se appears to be spared in patients if the stimulus is presented alone [33, 38], they have difficulty dissociating the BM signal from surrounding noise signals [33, 45] and discriminating BM from non-BM stimuli [33, 38, 46]. For instance, patients with schizophrenia show poor performance in detecting BM from noise elements [33], requiring a smaller amount of noise for comparable detection to healthy observers. Such impaired identification of BM is thought to be related with the different pattern of the late positive potential in a recent EEG study [46]. That is, the BM-specific processing in patients may not be as successful as that in unaffected individuals. It can also be seen through patients’ higher tendency to misperceive BM. Results from other types of tasks, mainly discrimination or recognition, have indicated that patients with schizophrenia tend to judge non-BM stimuli as BM or exhibit higher “false-alarm” rates in their responses [33, 38]. A functional magnetic resonance imaging (fMRI) result provided supporting neural evidence by showing similar activation between BM and non-BM within the posterior superior temporal sulcus (pSTS) in schizophrenia [38], where the pSTS is the region showing selectively strong activation to BM in the normal brain [47–51]. Taken together, BM, when presented in a WM task, would have an advantage in the encoding process compared to plain stimuli, such as geometrical shapes or random motions, only if its socially meaningful information were successfully decoded in perceptual processing, which in turn would facilitate encoding, like faces in other studies [52, 53]. Thus, we presumed that BM would be a useful stimulus for observing WM encoding efficiency in patients with schizophrenia in comparison with healthy people.

In the present study, we attempted to explore how perceptual processing affects WM encoding and examined accuracy in schizophrenics, compared with healthy controls. Specifically, considering previous findings of (1) a wide range of WM deficits in schizophrenia, (2) WM encoding being modulated by the salience of stimuli, (3) characteristics of BM perception in healthy individuals, and (4) impaired BM perception in schizophrenia (i.e., poor detection and tendency to misperceive), we designed a delayed-response task for measuring WM in which BM and two types of comparison stimuli (non-BM and static polygons) were used. Through the experiment, we observed how the encoding of BM stimuli, which is assumed to be different in healthy individuals and patients with schizophrenia in terms of perceived salience relative to other types of control stimuli, would be reflected in WM accuracy.

We hypothesized that healthy observers would show higher WM accuracy when BM should be remembered compared with the trials of non-BM and static polygons. BM could be precisely and rapidly perceived; in addition, its rich socially meaningful information could increase its salience. Therefore, it was assumed that healthy observers would benefit from BM in the WM task. In patients with schizophrenia, on the other hand, more complicated results were expected depending on their perception of BM. One possibility was that the patients with schizophrenia would exhibit a lack of difference in WM performance between BM and non-BM stimulus trials, as, according to previous studies, patients are not very successful in discriminating BM and BM-like scrambled motion (SM). There was another possibility: even though they do not seem to process BM as efficiently as healthy people do, they can still recognize BM as BM when the stimulus is presented alone without distracting noise signals. This implies that when the cognitive load is manageable in spite of the relatively inefficient BM process in schizophrenia, they may perceive the stimuli with fair precision. Thus, it was also possible that patients would take advantage of the perceptual salience of BM, resulting in slightly higher accuracy in BM trials compared to non-BM stimulus trials.

## Methods

### Participants

Nineteen patients who met DSM-IV criteria [1] for schizophrenia or schizoaffective disorder (9 males and 10 females) were recruited from a Community Mental Health Center in Seoul, Korea. Diagnoses were confirmed via the Korean version of the Structured Clinical Interview for DSM-IV (SCID) [54]. Clinical symptoms were assessed with the Korean version of the Positive and Negative Symptom Scale (PANSS) [55, 56]. The mean illness duration was 14.0 (S.D. = 10.21) years. Fifteen patients were taking antipsychotic medication at the time of testing, and the mean chlorpromazine equivalent (CPZ) doses [57, 58] of those patients was 279.40 (S.D. = 223.47) mg/day.

Twenty-six healthy controls were recruited from the local community in Seoul and from Duksung Women’s University. None of the controls had a history of drug or alcohol abuse, head injury, or mental disorders listed in DSM-IV Axis I/II. The Schizotypal Personality Questionnaire (SPQ) [59] was administered for the additional screening of people with high schizotypal personality scores. All controls scored well below the conventional cutoff score, which is 41 out of 74 [59].

The vocabulary subtest in the verbal comprehension section of the Korean-Wechsler Adult Intelligence Scale-Fourth Edition (K-WAIS-IV) [60] was administered to all participants. The two groups were matched in age and education level but differed in verbal IQ (p = 0.049). A summary of the demographic information is provided in Table 1. All participants had normal or corrected-to-normal vision, were provided with a detailed description of the procedure, and gave written consent. The study protocol was approved by the Institutional Review Board of Duksung Women’s University.

**Table 1:** Demographic information of participants.

	Patients (n = 19)	Controls (n = 26)	*p*
Gender (M/F)	9/10	9/17	
Age (year)	37.3 (10.5)^1)^	33.9 (11.6)	0.33
Education (year)	13.6 (2.1)	14.7 (1.8)	0.051
Verbal IQ (raw score)	41.6 (8.9)	45.8 (5.0)	0.049*
CPZ doses (mg)	279.4 (223.5)^2)^	n/a	
PANSS—positive	13.8 (5.1)	n/a	
PANSS—negative	13.4 (3.7)	n/a	
SPQ (raw score)	n/a	11.6 (9.8)	

^1)^ Mean (Standard deviation)

^2)^ CPZ doses were calculated from 15 patients who were taking medication.

### Stimuli

Three types of stimuli were used in the experiment—PL BMs, pairwise shuffled motions (PSMs), and polygons—and each type contained 48 stimuli (half of them were mirror-reversed ones of the other half). All stimuli were presented on the LCD screen an iMac computer (Apple, Cupertino, CA) running Matlab (MathWorks Inc., Natick, MA) and the Psychophysics toolbox [61, 62]. BM animations were generated using motion data provided by Carnegie Mellon University Motion Capture Database online and BioMotion toolbox [63]. BM stimuli consisted of 12 dots placed on the head and major joints of the body and depicted various actions, including throwing, kicking, walking, and jumping.

PSMs [64] were created by swapping six body parts (head-hip, shoulders, left and right arms, left and right legs) of a BM (each part consisted of two dots). Before shuffling the dot pairs, the mean locations of each dot throughout the temporal frames were calculated and then swapped with each other. Detailed rules and restrictions for creating PSMs are described in our previous study [64]. PSMs are similar to the spatially SMs, in that they disorganize the global motion of BMs while keeping the local dot trajectories the same, but they have an additional strength, because they also maintain the unique local features of BMs, such as pendular movements.

**Fig 1:**
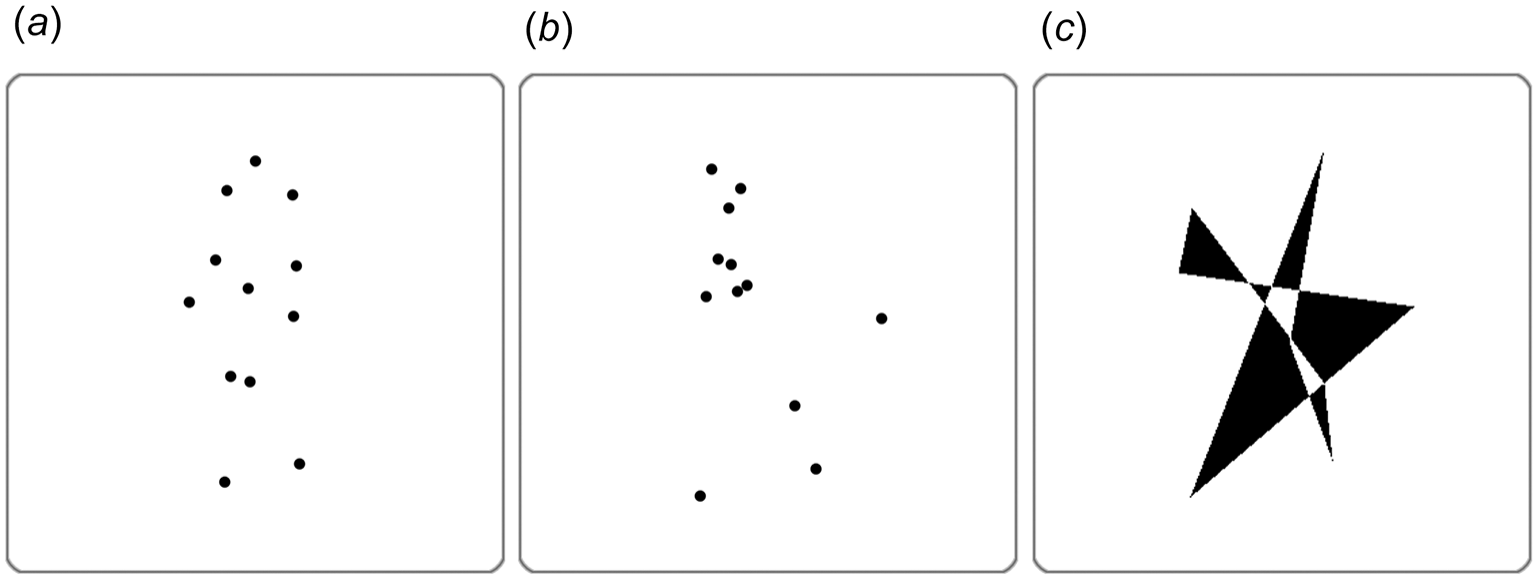
Examples of stimuli used in experiment. (A) BM. (B) PSM. (C) Polygon.

Polygons were produced by first generating a pool of eight random coordinate sets within the same range of motion stimuli and then selecting only those that were similar in size to the motion stimuli (Fig 1).

### Procedure

Although previous WM studies demonstrated that three to four visual items can be maintained at the same time [65–67] in healthy people, we found from our pilot test that it would be too difficult for schizophrenia patients to remember more than two items. Moreover, because we presented the stimuli serially, rather than simultaneously, it would increase the difference in terms of retention time between the first and the last item if we used more items. Hence, participants in the present study were asked to remember one or two items in each trial.

**Fig 2:**
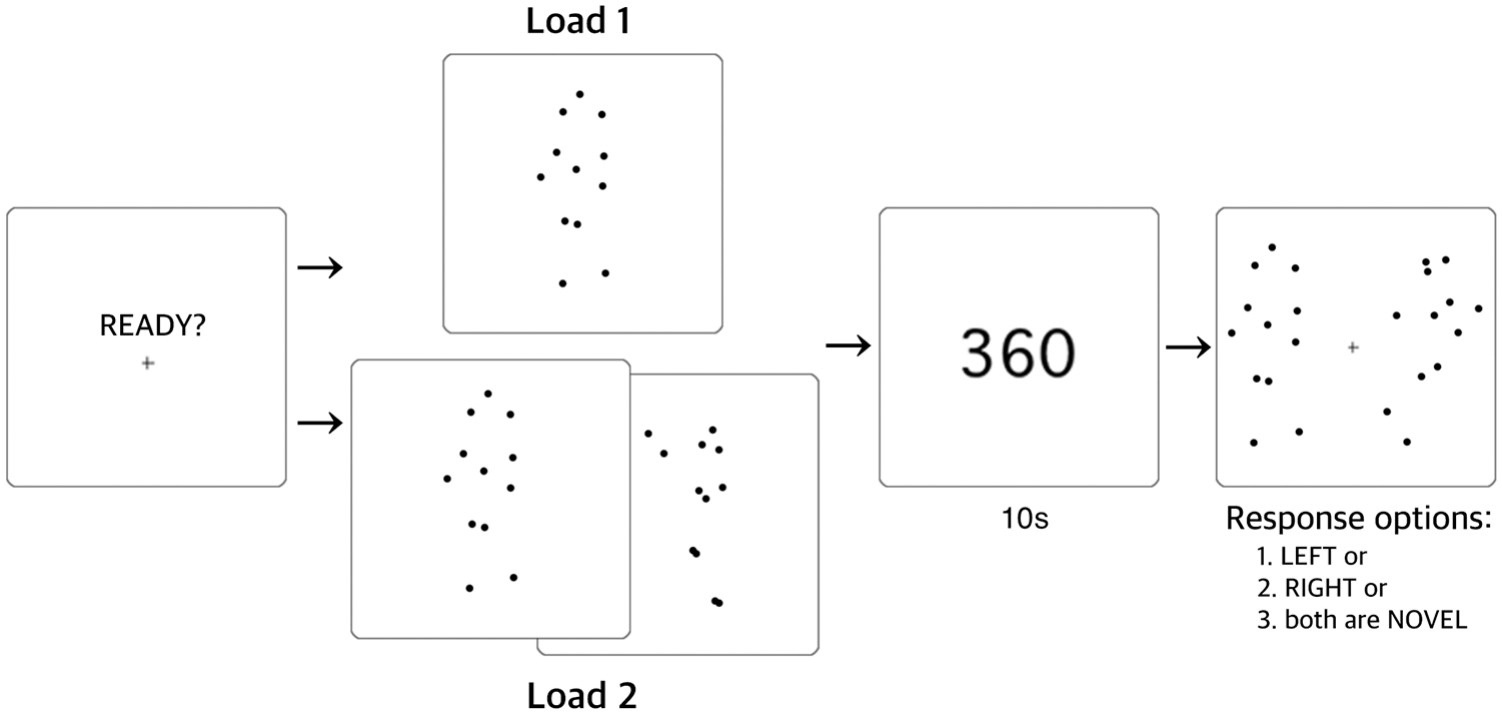
Structure of typical experimental trial. Example from BM block.

Each trial started with a ready screen on which there was a fixation cross it the center. Directly after a key press, the screen with the memory stimulus followed. In the Load 1 condition, participants were required to remember one item that was displayed for 1 s. In the Load 2 condition, two items were sequentially presented for 1 s each with an inter-stimulus interval of 1 s. The approximate size of the area occupied by each stimulus was 7 (width) x 9 (height)°. A backward counting task of a three-digit number was conducted as an intervening task for 10 s during the retention period to prevent general rehearsal and maintain sustained attention. A blank screen with a fixation cross was given for 1 s both before and after the intervening task, resulting in a total delay of 12 s between the memory stimulus presentation and retrieval. After the intervening task during the delay period, two different animations (BMs or PSMs) or polygons were provided on each side of the fixation cross, with the center of the figure being approximately 5° apart from the fixation cross. Response accuracy was measured by a three-alternative forced-choice: the participants pressed one of three designated keys to indicate whether the probe on the left was in their memory, the one on the right was in their memory, or both were novel. The probes were displayed until the participants responded. The numbers of trials in which the target was on the left or right or not probed were the same (33%). The experiment consisted of three separate blocks (BMs, PSMs, and polygons), and each block contained 48 trials. The order of the blocks was counterbalanced between participants, and the load size was counterbalanced within each block. A schematic illustration of a single trial is shown in Fig 2.

## Results

### Accuracy

#### Group difference and main effects of stimulus type and memory loads

A repeated measures ANOVA (stimulus type × load × group) on mean accuracy showed that the patients with schizophrenia were less accurate in recognition compared to the healthy controls. Overall mean accuracies (%) in patients and healthy controls were 54.36 (S.D. = 9.34) and 80.34 (S.D. = 9.34), respectively (F(1,43) = 85.04, p < .001). Another significant main effect was for stimulus type (F(2,86) = 40.95, p < .001). Post-hoc analysis indicated that BMs (M = 72.4) and polygons (M = 70.64) were easier to remember compared to PSM stimuli (M = 59.01). In addition, the accuracy difference between the Load 1 and Load 2 conditions was also significant (F(1,43) = 47.26, p < .001). The mean accuracy for the Load 1 condition was 71.77 (S.D. = 10.42), while that for the Load 2 condition was 62.93 (S.D. = 10.35).

#### Interaction effects

Interactions between any two variables were not significant (stimulus type × group: F(2,86) = 2.41, p = 0.09; stimulus type × load: F(2,86) = 0.32, p = 0.73; load × group: F(1,43) = 2.84, p = 0.1). However, the three-way interaction among these variables was significant (F(2,86) = 5.35, p < 0.01). This interaction was due to the distinctive pattern between groups observed in the Load 2 condition, as there was no significant interaction when the results from the Load 1 condition alone were analyzed (F(2,86) = .12, p = .89).

**Fig 3:**
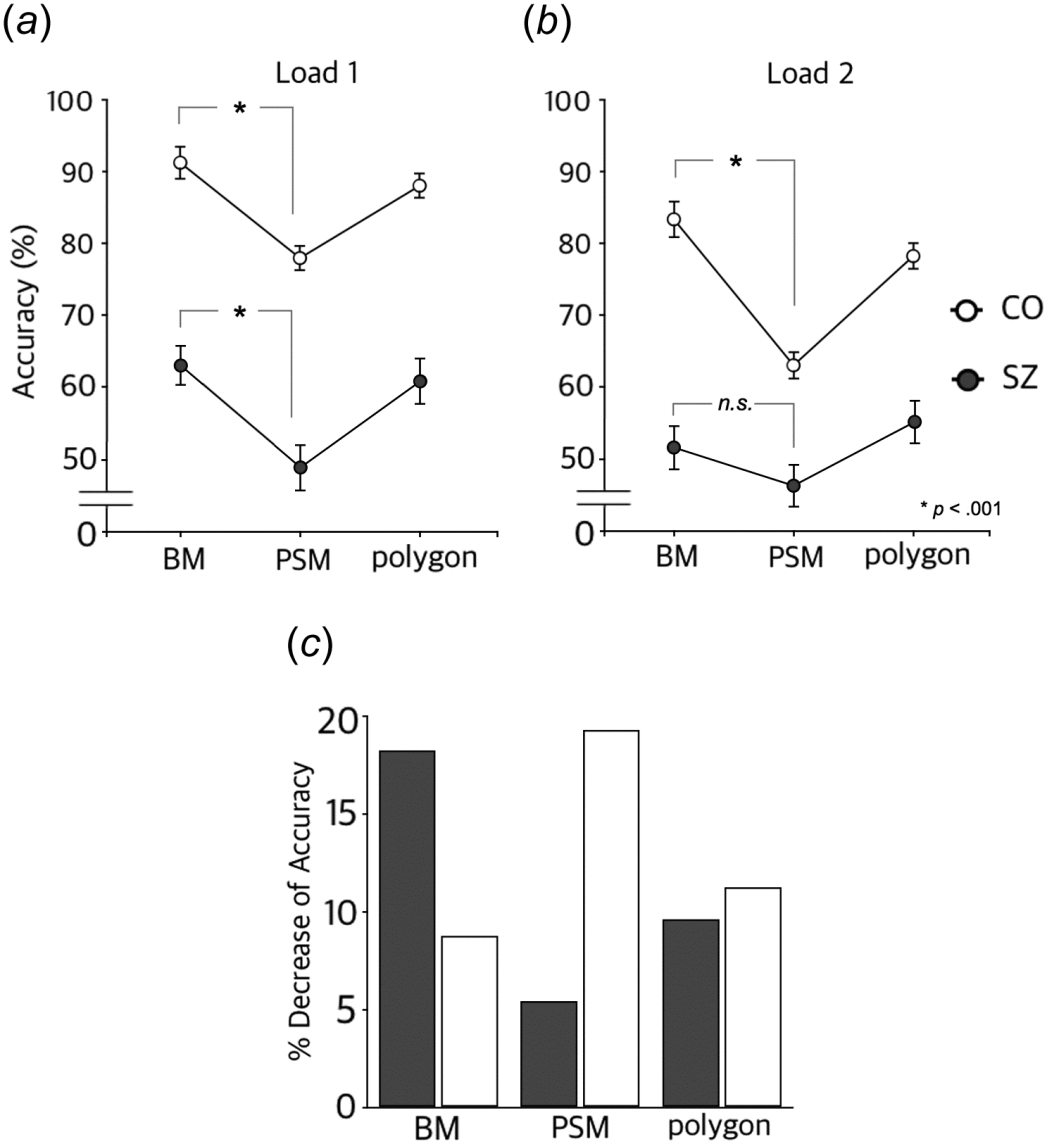
Experimental results. (A and B) Mean accuracy from Load 1 and Load 2 conditions in patients with schizophrenia (SZ) and healthy controls (CO). Error bars indicate standard error of the mean. (C) Percent decrease from mean accuracy in Load 1 condition to mean accuracy in Load 2 condition.

Specifically, the extent to which accuracy was affected by the increase in memory load differed between the two groups according to the type of stimulus, which can be easily noticed by comparing Fig 3A and 3B. To visualize the differences more clearly, the mean accuracy drops from Load 1 (Fig 3A) to Load 2 (Fig 3B) in each stimulus condition in the two groups are illustrated in Fig 3C. While mean accuracy in recognizing polygons in both groups dropped to a similar degree, the percent decrease in mean accuracy for motion stimuli (BMs and PSMs) showed a different pattern between the patients and controls. BM was the least affected by the memory load increase in healthy controls, which was consistent with the hypothesis that BM would facilitate encoding and benefit WM in individuals who could perceive it properly. In contrast, the memory performance for PSM showed a greater decrease with the memory load increase. The patient group exhibited the opposite pattern: the greatest decrease in memory performance in higher memory load was observed in BM trials, while the performance for PSM stimuli was the least affected by the manipulation of the memory load.

The significant difference that existed between BM and PSM in the Load 1 condition in the patient group disappeared in the Load 2 condition. However, the significant difference remained in healthy controls across both load conditions (Load 1: t(18) = 4.52, p < .001; Load 2: t(18) = 1.99, p = .063 for schizophrenia; Load 1: t(25) = 6.39, p < .001; Load 2: t(25) = 7.14, p < .001 for controls). The interaction effect of the repeated measures ANOVA became more robust between stimulus type × load × group (F(1,43) = 13.38, p < .001), and the stimulus type × group interaction reached a significance level (F(1,43) = 4.45, p < .05), while the load × group interaction was not significant (F(1,43) = 1.68, p = .20) when only BM and PSM were considered.

Although chance-level performance for the task was 33% correct, and the mean accuracies of both groups in each load condition were higher than the chance level, concerns can be raised as to whether the lack of difference between BM and PSM in the patient group in the Load 2 condition was driven by a possible floor effect. Indeed, a number of participants in the patient group failed to perform significantly above chance level. We conducted extra analyses after excluding data from those who performed at near-chance levels to scrutinize the possibility. When only those who scored above 33% for both BM and PSM were considered, the difference between BM and PSM in the Load 2 condition in the patient group still did not reach the significance level (t(12) = 2.00, p = .069). Even when we applied a stricter cutoff (above 43% accuracy, which is significantly higher than 33% according to chi-square), the result remained the same (t(7) = 2.08, p = .077). Hence, the lack of difference between BM and PSM in the Load 2 condition in the patient group did not necessarily seem to be driven by the subjects who performed at near-chance levels.

### Response time (RT)

A three-way repeated measures ANOVA on RT also revealed main effects of stimulus type (BM: 3412ms, PSM: 4370ms, polygon: 2566ms, F(2,86) = 53.79, p < .001) and memory load (Load 1: 3131ms, Load 2: 3768ms, F(1,43) = 81.17, p < .001). Group difference, however, was not significant (controls: 3375ms, patients: 3524ms, F(1,43) = .19, p = .66). Moreover, unlike the accuracy results, there was no significant three-way interaction effect (F(2,86) = 1.66, p = .20).

When RTs for BM and PSM were compared, the healthy control group exhibited differences between the two stimulus types in both load conditions (BM: 2739ms, PSM: 3868ms, t(25) = 6.76, p < .001 for Load 1; BM: 3716ms, PSM: 5082ms, t(25) = 4.61, p < .001 for Load 2). However, the patient group did not (BM: 3423ms, PSM: 4091ms, t(18) = 1.62, p = .12 for Load 1; BM: 3770ms, PSM: 4438ms, t(18) = 2.04, p = .056 for Load 2). Taken together, the RT for BM was faster than that for PSM in the control group, regardless of memory load. On the other hand, the patient group did not show a significant difference between BM and PSM trials in either load condition.

### Relationship between task performance and demographic variables

To determine if any of the demographic or clinical variables might be related with the results from the WM tasks, we conducted correlation analyses. None of the variables (PANSS, SPQ, illness duration, CPZ doses) was significantly correlated with memory accuracy, implying that the performance differences were less likely to be attributed to clinical variables. Across all participants, IQ score showed a significant positive correlation with accuracy for BMs and polygons in the Load 1 and 2 conditions, and accuracy for PSMs in the Load 1 condition. However, within each group, IQ score was not significantly correlated with accuracy for any stimulus types in either load condition.

## Discussion

In the present study, we investigated WM in patients with schizophrenia and healthy controls. We used PL BM as the main stimulus and examined if this type of motion stimulus would have a facilitating effect for encoding in WM compared with other types of stimuli (i.e., PSMs and polygons in the present study). Overall, the patients with schizophrenia exhibited significantly lower WM accuracy across all types of stimuli regardless of the level of memory load, which indicated impaired WM in schizophrenia, and this finding is consistent with ample reports from previous studies.

As noted in the introduction, we hypothesized that WM accuracy for BM trials would be higher than that for other types of stimuli in healthy controls. Conversely, we expected two possible results from the patients with schizophrenia depending on their earlier processing of BM. We expected that they would also exhibit higher accuracy for BM trials compared with non-BM trials or that there would be a lack of accuracy difference between BM and non-BM trials due to their less precise discrimination ability. The results from the healthy controls supported our hypothesis, in that the mean WM accuracy for BM was higher than that for non-BM stimuli. In patients with schizophrenia, interestingly, we observed both possible results we assumed in each load condition, thus yielding the significant three-way interaction among group, stimulus type (especially between BM and PSM), and number of memory items (i.e., memory load). The patients with schizophrenia exhibited higher WM accuracy for BM compared with PSM, like controls, when there was only one target to be remembered (Load 1). However, this pattern was no longer observed in the Load 2 condition in which the accuracies for BM and non-BM were comparable. We would like to focus on this BM-facilitating effect on WM accuracy in each group in the following paragraphs.

### Healthy controls

In healthy controls, WM accuracy for BM stimuli was superior to that for PSM stimuli for both the Load 1 and Load 2 conditions. As mentioned earlier, previous WM studies have reported that stimuli novelty or salience could facilitate the encoding process, resulting in greater WM accuracy in retrieval [21]. Moreover, according to perceptual studies, BM is a unique motion stimulus that is almost effortlessly perceived [41]. Thus, it appears reasonable to conjecture that BM could also work as another salient visual stimulus based on its unique pendular motion expressing familiar human action with rich social information. In addition, each BM stimulus depicted a specific human action likely to be related to meaningful cause-effect information, which could have facilitated deep processing and thus benefited the encoding process. For instance, a PL BM depicting a punching action may have triggered an instantaneous thought, consciously or not, about what the agent was hitting or how damaged the object would be. As shown in Fig 3A and 3B, mean accuracy for BMs was significantly greater than that for PSMs, suggesting that the BM facilitation effect affected encoding in WM processing as hypothesized.

One thing to be considered is the result from the polygon trials. As described in the methods section, the polygons in this study were randomly generated, thus providing no meaningful information. Accuracies for polygon trials in both memory load conditions were slightly lower than those in BM trials, but the difference was negligible. We presume that the polygon trials may have been easier than the PSM trials. These two types of stimuli are similar, in that they are both randomized, disorganized, and meaningless. In the case of PSM, however, the encoding load would be much greater than that for polygons considering the fact that PSM was not a single static frame but rather had additional motion information. Thus, we speculate that the accuracy difference between the PSM and polygon trials in both memory load conditions reflects this difference in difficulty. In this context, it is tempting to argue that BM, regardless of its complicated nature in terms of the motion information, benefits encoding due to its innate rich meaningful/social information, and therefore, it could not only compensate for the expected decrease in performance but also even resulted in the highest accuracy among all stimulus types.

### Patients with schizophrenia

The patients with schizophrenia showed an analogous response pattern to the healthy controls in the Load 1 condition, although the overall accuracies across the stimulus types were lower compared to controls. These results suggest that schizophrenia patients also have more difficulty encoding motion stimuli (i.e., PSM) than meaningless static polygons and that BM stimuli have facilitating effects on WM processing, as in the healthy controls.

However, when the memory load increased (Load 2 condition), the accuracy pattern of patients with schizophrenia across the stimulus types differed from that of the healthy controls. As the memory load increased, unlike with controls, the BM facilitation effect disappeared (Fig 3B): in the Load 2 condition, the accuracy of BM trials was not significantly higher than that of PSM trials in schizophrenia patients. Furthermore, encoding PSM could have been relatively more effective in the patient group, because the accuracy for PSM trials did not decrease significantly with the higher memory load, as seen in Fig 3C.

Taken together, a BM facilitation effect on WM also exists in schizophrenia patients, but it seems to be vulnerable to increasing memory load. In healthy controls, the effect of the efficient encoding of BM is solid enough to be maintained for at least two serially presented stimuli, which is remarkable considering the dropped accuracy (Fig 3C) for another type of stimulus with physical features similar to those of BM but without any meaning (i.e., PSM). On the other hand, the patients with schizophrenia might have experienced overload of BM encoding with increasing memory load. That is, interference might have occurred between the two BMs otherwise processed effectively, thus leading to comparable accuracy between BM and PSM trials in the Load 2 condition.

There are some additional potential explanations for our results in terms of possible abnormalities in the perceptual and encoding phases in WM in schizophrenia. Previous perceptual findings indicated that the ability to recognize BM per se is spared when BM is presented alone [33, 37]. Thus, the patients might have encoded BM properly in the Load 1 condition in the present study, which could have been reflected as the BM facilitation effect, similar to the pattern shown in healthy controls. However, when the patients were asked to discriminate BM and non-BM, or to detect BM from noise elements, they showed significantly lower accuracy [37, 38], suggesting inefficient perceptual processing for more than one BM stimuli or BM with noise concurrently. Thus, it is plausible that the patients also benefit from BM in WM, although this effect may be susceptible to WM load because of their limited, inefficient perceptual processing.

### Other considerations

#### RT

Although RT was not of our main interest and not considered in our hypotheses, RT is another important index for the performance of perceptual and cognitive tasks: thus, we analyzed RT as well. Our RT analyses did not show a significant difference between groups, unlike the accuracy results. RTs from BM and PSM trials, however, showed different patterns across the groups: the healthy controls responded faster in BM trials compared with PSM trials, while the RT difference was negligible in the patients, regardless of the number of memory items. The results, together with the accuracy results, suggest that BM was more efficiently remembered than PSM in healthy controls but not in the patients despite BM being more accurately stored than PSM in the patients as well in the Load 1 condition. However, the interpretation should be considered with caution, because we did not put as much emphasis on RT as on accuracy when giving the task instructions to the participants.

#### Limitations and suggestions for future research

There are potential caveats and limitations to be considered along with future suggestions in the present study. First, most patients were taking antipsychotic medication at the time of testing. We do not entirely exclude the possibility of a medication effect, but we did not observe any significant correlations between CPZ doses and other variables, including WM performance. Second, our design might have underestimated participants’ WM capability. Although we presented two stimuli at the encoding stage, there were three response alternatives at the retrieval stage, which might have placed an additional processing load on all participants, and the schizophrenia patients could have been more impacted. Possible future research includes a similar paradigm in which the load in the response stage is much less. Third, we in part overlooked the fact that BM stimuli, unlike PSMs and polygons, could be named or verbally labeled according to the meaning of the motion, which possibly facilitated the encoding of the stimuli. It is true that BM was the only meaningful stimulus type among the three types of stimuli we used and that it might be problematic with regard to a putative imbalance in task difficulty among the stimuli. However, we are suspicious of the impact of the so-called naming effect on the results considering the accuracy drop for BM according to the load increase in the patient group. Assuming that the naming effect was crucial, it remains unclear as to why the patients did not seem to benefit from the naming strategy in the Load 2 condition, unlike in the Load 1 condition. In addition, if participants were highly dependent upon the naming strategy, the accuracy for BM should have been much higher compared with the other types of stimuli because the number of memory items was only two at most. This was not the case, as accuracy differences between BMs and polygons were negligible across all conditions. The introduction of the intervening task also minimized the effect by precluding verbal rehearsal. Thus, even though BM can be labeled due to its meaningful nature, the magnitude of the contribution of the naming effect seems limited, and the BM facilitation effect is not merely driven by the naming strategy. Lastly, our study was the first to use BM to explore whether a perceptual advantage caused by the salience and meaningfulness of the stimulus can affect WM performance, and it was successful in providing a clue that this effect is valid but may be vulnerable in schizophrenia. However, it is limited, in that we have not directly analyzed the relation through regression. A follow-up study focusing on a more direct measure to elucidate the relation would be valuable.

## Conclusion

In sum, we explored how WM in schizophrenia is modulated for a stimulus that patients may or may not find to be more salient than others by employing a unique dynamic stimulus (i.e., BM). The results of the current study revealed the possibility that patients with schizophrenia, as well as healthy individuals, are able to benefit from the perceptual salience of a socially relevant memory stimulus and provided an insight that the special effect may depend on the cognitive load placed upon patients.

## Supporting information

S1 Dataset Dataset for analyses. (XLSX)
